# Flavivirus–Host Interaction Landscape Visualized through Genome-Wide CRISPR Screens

**DOI:** 10.3390/v14102164

**Published:** 2022-09-30

**Authors:** Aditi Kanojia, Mansi Sharma, Rishad Shiraz, Shashank Tripathi

**Affiliations:** 1 Centre for Infectious Disease Research, Indian Institute of Science, Bengaluru 560012, India; 2 Microbiology & Cell Biology, Indian Institute of Science, Bengaluru 560012, India

**Keywords:** genome-wide CRISPR screens, flaviviruses, virus-host interactions

## Abstract

Flaviviruses comprise several important human pathogens which cause significant morbidity and mortality worldwide. Like any other virus, they are obligate intracellular parasites. Therefore, studying the host cellular factors that promote or restrict their replication and pathogenesis becomes vital. Since inhibiting the host dependency factors or activating the host restriction factors can suppress the viral replication and propagation in the cell, identifying them reveals potential targets for antiviral therapeutics. Clustered regularly interspaced short palindromic repeats (CRISPR) technology has provided an effective means of producing customizable genetic modifications and performing forward genetic screens in a broad spectrum of cell types and organisms. The ease, rapidity, and high reproducibility of CRISPR technology have made it an excellent tool for carrying out genome-wide screens to identify and characterize viral host dependency factors systematically. Here, we review the insights from various Genome-wide CRISPR screens that have advanced our understanding of Flavivirus-Host interactions.

## 1. Introduction

The family *Flaviviridae* constitutes many enveloped single-stranded positive-sense RNA viruses. This RNA genome encodes a single open reading frame that is translated at the Endoplasmic Reticulum (ER) to give rise to a polyprotein, which is subsequently cleaved by viral and host cell proteases. This processing forms ten functional proteins, including the three structural proteins, Capsid (C), Pre-membrane (prM), and Envelope (E), and seven non-structural proteins, which include NS1, NS2A, NS2B, NS3, NS4A, NS4B, and NS5. The family *Flaviviridae* consists of three viral genera, Flavivirus, Pestivirus, and Hepatitis C virus (HCV), with a total of more than 70 viruses, most of which have their polyproteins organized in a similar way. NS1 has a role in replication [1], as well as innate immune evasion by interfering with the Toll-Like Receptor (TLR) signaling pathway [2]. NS2A has a direct role in the replication of viral RNA. NS3 serves as the helicase and also interacts with NS2B as a cofactor (NS2B3) to form the viral protease [3,4]. NS4B inhibits the interferon (IFN)-dependent signaling pathway [5]. NS5 is both the RNA-dependent RNA-polymerase, which catalyzes genome replication and methyltransferase, which caps the nascent RNA genomes [6,7]. In addition to these, the viral RNA genome also contains 3′ and 5′ untranslated regions (UTRs) that contribute to genome stability and translation [8]. Flavivirus genome replication occurs within ER membrane involuted structures called virus replication compartments (RCs) [9]. These remodeled ER substructures assist in concentrating the replication substrates and shielding the viral RNAs from detection by the host immune system. NS4A protein is the key organizer of these ER membrane structures [10]. Within these RCs, the NS5 protein, along with other viral and human host factors, performs the enzymatic steps of genomic viral RNA replication [11]. The components then assemble and bud off from the ER through the trans-Golgi pathway and are trafficked out of the cell via exocytosis. The maturation step is associated with the cleavage of prM by viral and host furin-like proteases. Some features that differ among the three genera include the existence of an additional cleavage site in the NS5 region of HCV and pestiviruses, but not flaviviruses. This site separates the N-terminal portion (NS5A) from the viral polymerase (NS5B) [12].

There are 53 defined species in the genus *Flavivirus*. Some medically important flaviviruses are associated with a spectrum of diseases ranging from mild self-limiting febrile illness to severe life-threatening encephalitis, hepatitis, vascular shock syndrome, congenital abnormalities, and Viral hemorrhagic fevers (VHFs). These include Dengue (DENV), Japanese Encephalitis (JEV), West Nile (WNV), Zika (ZIKV), and yellow Fever (YFV) viruses. Currently, DENV fever is the most prevalent arthropod-borne viral disease globally, with an estimated 390 million total infections, 100 million clinically apparent cases, and 500,000 presentations of severe dengue per year worldwide, with at least 2.5 billion people at risk [13]. ZIKV has been correlated with severe congenital abnormalities such as microcephaly and other birth defects in unborn children due to potential ZIKV exposure to the mother during pregnancy [14]. JEV, which primarily affects children, is estimated to cause approximately 14,000 to 20,000 fatal cases annually [15]. WNV virus is maintained in nature in a mosquito–bird–mosquito transmission cycle but has spilled over and caused disease in humans and horses. The spread of WNV and JEV across different geographical locations has been attributed to the seasonal migrations of birds [16].

Most of the viruses in the genus *Flavivirus* survive in nature by replicating alternately in a vertebrate host and a hematophagous arthropod (mosquitoes and ticks) and hence are classified as arboviruses. These arthropod vectors acquire the virus by biting a viremic host. The virus then replicates in the vector’s tissues. The transmission to another vertebrate host happens through salivary secretions of these arthropod vectors as they take up blood meal. The virus then multiplies within the vertebrate host, causing viremia and disease. Non-human primates, mostly wild mammals and birds form the principal vertebrate hosts for most flaviviruses, thus leading to the sylvatic transmission cycle [17]. The natural zoonotic cycle of these viruses does not usually involve humans. However, a few viruses can jump into a human–mosquito–human cycle. This is called the urban cycle, where humans catch the disease when they encroach on forest habitat and are bitten by the arbovirus-infected mosquitoes. When these people move to densely populated urban settings, such infections are transmitted by highly anthropophilic urban mosquitoes and can lead to explosive outbreaks. This is when a sylvatic transmission cycle is said to have ‘spilled over’ into an urban transmission cycle. An excellent example is the emergence of sylvatic YFV during an epidemic in the Gambia [18,19]. Thus, because of their high transmission potential, these viruses can cause severe and widespread outbreaks in many tropical and subtropical regions of the world, depending upon the presence of appropriate insect vectors.

Since most of the flaviviruses are vertically transmitted in the arthropods, they may elicit long-term persistence and potential re-emergence. Therefore, improving or coming up with novel strategies to blunt flavivirus disease is of the essence, even if better vaccines or antivirals become available. The cellular interactions of flaviviruses with their human or mosquito hosts are critical for manifesting the viral life cycle and decoding this information can be very useful for engineering novel strategies to disrupt the disease or its transmission. Genome-wide CRISPR knockout (GeCKO) screens offer an exceptional approach to identifying such host factors. The revelation and validation of these host dependency factors can be followed by discovering or designing small molecule drugs to suppress them. It is important to note that therapeutic intervention of host factors, rather than viral proteins, is associated with a much higher barrier to drug resistance. In recent years, GeCKO screens have successfully identified host dependency factors of several clinically relevant viruses other than Flaviviruses [20]. These include Influenza A [21,22], Coronaviruses [23,24], picornaviruses [25], and HIV [26]. Moreover, understanding these interactions has paved the way for developing efficient therapeutics and preventive strategies against these infections [27,28].

This review gives a brief overview of GeCKO Technology and summarizes various studies that have utilized it to identify host dependency and restriction factors for Flavivirus Infection.

## 2. CRISPR-Cas Biology: An Overview

The clustered regularly interspaced short palindromic repeats (CRISPR)-Cas system was initially discovered as a sophisticated acquired immune system in Prokaryotes and Archaea, where it acts to protect against invading bacteriophages and conjugative plasmids by degrading their genetic elements [29,30]. At the molecular level, there are two key players in this process: the CRISPR RNA (crRNA) and its associated endonuclease (Cas) [31]. CRISPR systems have been rapidly implemented to perform eukaryotic genome manipulation. As seen in their natural prokaryotic counterparts, the engineered CRISPR systems also have two effector molecules: a single guide RNA (sgRNA) chimera, consisting of a fusion of a crRNA and tracrRNA, and a Cas protein. The sgRNA comprises a customizable spacer sequence of ∼20 nucleotides and a scaffold sequence that binds to the Cas protein. The spacer can be designed according to the genomic target to be modified. Therefore, by merely altering the spacer sequence, the sgRNA-Cas Ribonucleoprotein complex can be directed to target any genetic loci [32,33]. For knocking out a gene, the sgRNA designed specific to the targeted gene and a Cas enzyme are made to co-express within the chosen cell type or organism. Upon binding to the target, Cas9 undergoes a conformational change and cleaves the opposite strands of the target DNA, creating a blunt-ended Double-strand break (DSB). When cellular repair mechanisms try to ligate the broken ends, they end up introducing small nucleotide insertions or deletions (indels) or frameshift mutations, leading to premature stop codons within the ORF of the target. This ultimately leads to a loss-of-function mutation within the targeted gene.

One can use the same sgRNA library combined with certain derivatives of Cas9 for programmable genetic manipulations [34].

**Figure 1:**
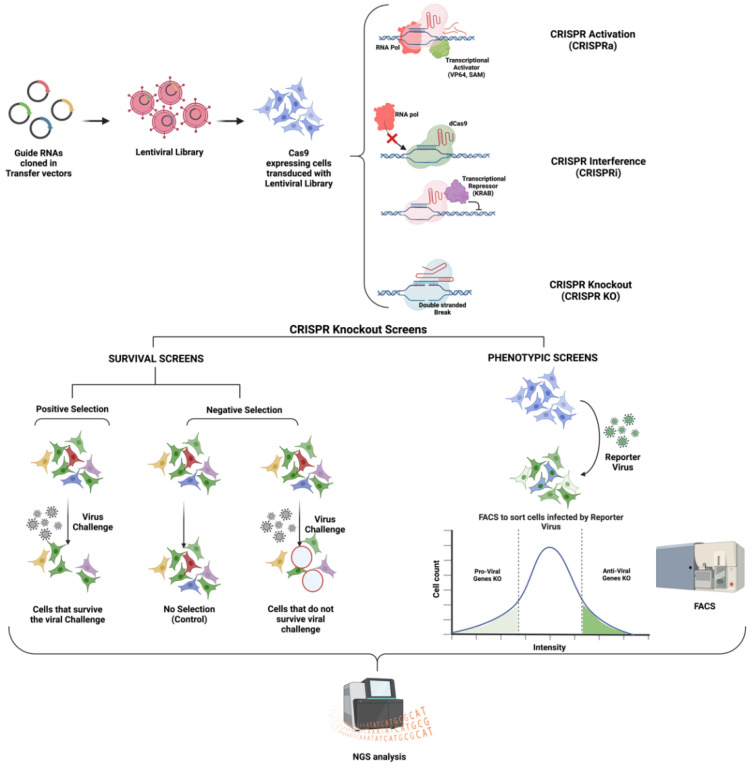
Schematic of different types of CRISPR Screens to identify virus-host interactions (Created with BioRender.com).

(1) A catalytically dead Cas9 enzyme (dCas9) for CRISPR interference (CRISPRi) studies: This dCas9 binds to the target DNA sequence guided by the gRNA. Instead of cleaving the bound DNA, the dCas9 enzyme remains bound to the target DNA sequence, disrupting RNA polymerase or transcription factor binding to the promoter. Other than steric hindrance, CRISPRi can also repress transcription via a repressor domain, such as the Krüppel associated box (KRAB), fused to dCas9 [35,36] (Figure 1).
(2) Cas9 tethered with a transcriptional activator such as SunTAG [33], Synergistic Activation Mediator (SAM) [37], VP64 [38], etc., for CRISPR activation (CRISPRa) studies: Such Cas9 leads to the recruitment of transcriptional machinery to the targeted promoter. CRISPRa studies are employed to perform gain-of-function studies [35,39].

In general, the idea of a genome-wide loss-of-function screening is to generate a large population of cells with a wide variety of genes knocked out or knocked down and then identify the genetic perturbations that lead to the desired phenotype, such as the survival of cells despite a lethal viral infection [20]. Presently, the most popular method for conducting GeCKO screens involves using pooled lentiviral CRISPR libraries, which is briefly elucidated in the next section and illustrated in Figure 1.

### Pooled Lentiviral CRISPR Libraries

These libraries consist of thousands of Lentiviral transfer plasmids, each containing a sequence encoding for a specific gRNA targeting a genetic locus in the host cell genome. A CRISPR library typically includes ∼3–6 independent gRNA sequences per gene to ensure high on-target and low off-target activity.

1. The guide RNAs are designed in silico based on the target sequence, synthesized using oligo arrays, and cloned in a pooled format in the lentiviral transfer plasmids. Several CRISPR libraries have been designed for applications such as genetic knockout, activation, and repression of all or specific classes of human genes. Different libraries may utilize different algorithms for gRNA designing and specificity analysis.
2. The next step is to package these plasmids into lentiviral vectors. These lentiviral vectors are then used to transduce the target cell line at a low virion-to-cell ratio, also called the Multiplicity of Infection (MOI). Low MOI ensures that there is only a single lentiviral infection per cell; therefore, only a single sgRNA is expressed per cell. Usually, transduction of the sgRNA library is performed at an MOI < 0.3 to ensure that a majority of the cells receive at most one genetic perturbation.
3. The cells with genetic integrations are enriched using drug selection or fluorescence-based cell sorting. This leaves one with a heterogeneous population of cells with a single gene knocked out per cell.
4. Next, selection pressure is applied to identify genes that either favor or suppress the desired phenotype. Screens can be of the following types:
  - Survival (KO) Screens: In positive survival screens, the cells that carry specific sgRNAs are selected. For example, an intense selective pressure, such as a cytolytic viral infection, will kill most cells except for a subset. These survivors will not show any cytopathic effects because of the knockout of host factors that support any of the events of viral pathogenesis: viral entry, translation, genome amplification, packaging, or virus-induced cell death [20,40]. In negative screens, the idea is to identify the cells that do not survive the selection pressure. Negative screens are often used to identify essential genes where the loss of function cannot support cell survival.
  - Phenotypic screens: Alternatively, one can perform a phenotypic screen using fluorescence-activated cell sorting (FACS). This relies on using genetically engineered reporters that carry a GFP, luciferase, or any other reporter gene that enables the quantification of events such as viral entry and replication. The strength of the fluorescence signal correlates with the viral load within the cell. Thus, the FACS assay can reveal the antiviral and pro-viral host genes [26,41].
  - In addition to knockout, some screens might involve activation or knock-down of the target genes:
  - CRISPRa (Activation) Screens: For identifying the essential genes’ role, if any, in the viral life-cycle, one can perform a CRISPR-mediated activation or a gain of function study to determine if ectopic overexpression of any essential host factor modulates the viral biology [28].
  - CRISPRi (Interference) screens: These screens are essentially based on knockdown of the target gene and utilize either dCas9 for a steric block of transcription or dCas9 associated with transcriptional repressor (KRAB) [35,39,42].
5. Finally, Next-Generation Sequencing (NGS) analysis is used to determine the genes that are either enriched or depleted compared to the control population.

Notably, a CRISPR screening experiment aims to narrow down a broad hypothesis. The genes identified in a screen need to be investigated and validated further to confirm that they do cause the biological effect under consideration.

Utilizing lentiviral vectors for delivering the gRNA comes up with certain limitations. Firstly, it is impossible to control where the viral genome will integrate into the host genome. This integration may disrupt the essential functions of the cell. This also implies that we cannot use the knockout approach to assess the genes essential for cell survival. Thus, CRISPR approaches that modulate the transcription levels of the genes (CRISPRa or CRISPRi) rather than leading to complete knockout offers a significant advantage by allowing us to study essential genes, as they can decrease gene expression without eliminating it [39]. Additionally, since the DNA integrates into the host genome, lentiviral delivery leads to long-term expression of Cas9, potentially leading to off-target effects (OTEs) and double-stranded breaks. Several alternate versions of Cas9 with enhanced on-target activity (such as HypaCas9 [43] and Sniper Cas9 [44,45]) have been developed to minimize oncogenicity and toxicity [46]. Importantly, the CRISPRi approach does not lead to the toxicity of active Cas9 and, therefore, allows for the silencing of noncoding RNAs and regulatory regions. Fusing dCas9 to the KRAB domain has been shown to potentiate CRISPRi in specific human cells [47]. CRISPRi suffers from incomplete knockout of the target gene and, therefore, cannot be used for genes that have a phenotype only on complete knockout [39].

Simple fusions of dCas9 to an activator domain such as VP64 helped achieve only modest activation of the gene of interest while using a single sgRNA [48]. To check this, CRISPRa constructs that recruit more than one activator domain with a single sgRNA have been developed [39]. However, both CRISPRi and CRISPRa warrant empirical information about the transcription start sites and positioning of the sgRNA to the transcription start site. Moreover, in conventional pooled CRISPR screens, specific rare cellular phenotypes may become very difficult to identify. Only crude or basic phenotypes such as cell survival or reporter gene expression can be recognized. Another prime consideration is the cell line since viruses differ in their host range and tissue tropism. To ensure the appropriate representation of each of the sgRNAs in the pool, numerous cells need to be transduced and undergo phenotypic selection. In practice, primary cells have a limited proliferative capacity [20]. Therefore, it is more challenging to transduce and expand these cells in large numbers. The difficulty in propagating certain viruses *in vitro* in cell cultures becomes a major limitation while trying to discern the molecular details of host-virus factors. In addition, in a pooled screen, the selection must be extremely stringent to ensure that only the perfectly resistant cells survive multiple rounds of infection. Thus, strong selection conditions causing marked phenotype are chosen. Although this high stringency helps identify high-confidence candidate genes (such as virus entry receptors), other genes that have relatively milder effects on infection may be missed out [20]. To check this, one can decrease the stringency or use naturally attenuated virus strains. However, such fine-tuning at the level of stringency is not always achievable in pooled screens. In such a scenario, arrayed screens may be a good alternative. Additionally, to decode the host factors that are required at specific stages of the viral life cycle, such as virus entry, genome replication, and translation, one can make use of pseudotyped viruses (viruses or viral vectors that are packaged with envelope proteins from another virus) [49], viral replicons (self-replicating subgenomic viral RNAs) [50], and internal ribosome entry site reporters (IRES reporters), respectively, during the infection assays [20].

Moreover, in conventional pooled CRISPR screens, certain rare cellular phenotypes may be very hard to identify, and only crude phenotypes such as cell survival, proliferation, reporter gene expression, etc., can be recognized. Another important consideration is the cell line since viruses differ in their host range and tissue tropism. To ensure the appropriate representation of each of the sgRNAs in the pool, numerous cells need to be transduced and undergo phenotypic selection. In practice, primary cells have a limited proliferative capacity, and it is therefore more challenging to transduce and expand these cells in large numbers. In addition, in a pooled screen, the selection needs to be extremely stringent to ensure that only the resistant cells survive multiple rounds of infection. Thus, strong selection conditions causing marked phenotype, where >99% of cells die from infection, are preferred. Although this high stringency increases the confidence in the candidate genes identified, other genes that have subtler effects on virus infection may be missed. Therefore, pooled screens for virus-host interactions tend to be biased towards the genes necessary for virus entry. To check this, one can decrease the stringency or make use of naturally attenuated virus strains. However, such fine-tuning of the stringency is not always possible in pooled screens. In such a scenario, arrayed screens may be a good alternative. Moreover, to identify host factors that are required at specific stages of the viral life cycle, such as virus entry, genome replication, and translation, one can make use of pseudotyped viruses (viruses or viral vectors that are packaged with envelope proteins from another virus), viral replicons (self-replicating subgenomic viral RNAs), and internal ribosome entry site reporters (IRES reporters), respectively, in the virus infection assay. Pooled approaches have been successfully employed to understand viral–host interactions. In a 2017 paper, Joung et al. offer a comprehensive, step-by-step protocol for different types of CRISPR screening [51].

## 3. CRISPR Screens for Studying Flavivirus Infections

Viruses, being obligate intracellular parasites, are intimately associated with their host cells [52]. While dual-host flaviviruses can cause disease in vertebrates as well as arthropods, insect-specific flaviviruses are restricted to their competent arthropods. How flaviviruses establish persistent infection in their insect vectors and humans relies upon an intricate interplay between flavivirus-encoded immune antagonists and the host antiviral innate immune effectors. In general, flaviviruses have a conserved replication cycle, which includes the following steps: viral entry via receptor-mediated endocytosis, fusion with the endosomal membrane and release of viral RNA, genome replication and translation into proteins in the ER membrane structures, virion packaging and processing through the trans-Golgi secretory pathway, and release of viruses via exocytosis. At every step, flaviviruses rely on the host machinery to facilitate replication, dampen host immune response, or disrupt cellular processes to aid pathogenesis. These host factors include proteins, RNAs, lipids, carbohydrates, or small molecules. They can be recognized by making use of techniques that probe direct or indirect physical interactions with viral RNA or proteins or through genetic interactions by perturbing the host, as in CRISPR and RNAi screens.

As mentioned before, CRISPR screens provide a tremendous advantage for high-throughput analysis of viral and host factors. Moreover, CRISPR-Cas technology is reliable for validating candidate genes. In contrast to gene knockdown approaches, such as RNAi, gene knockouts are absolute. They are associated with lesser variations when virus replication is quantified using assays such as qPCR, plaque assays, or immunostaining. Various genetic screens have been attempted over the last two decades to determine the host dependency and antiviral factors for multiple flaviviruses (Table 1).

**Figure 2:**
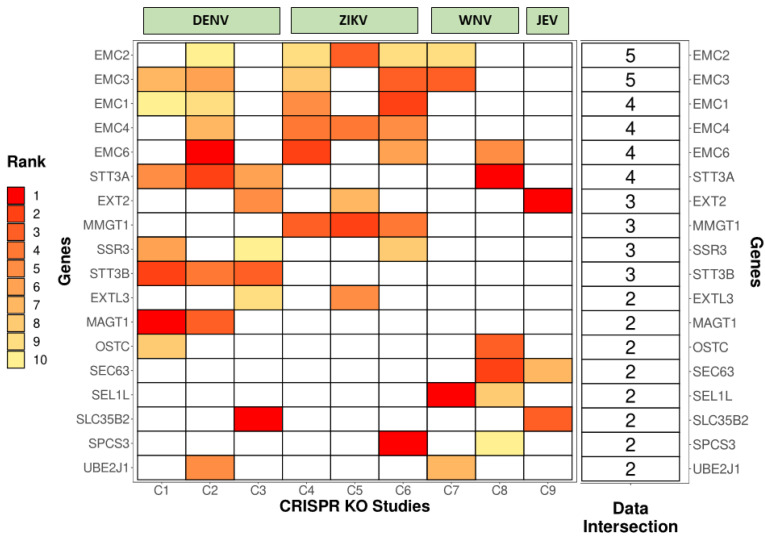
Intersections among different CRISPR KO studies for Dengue Virus (DENV), Zika Virus (ZIKV), West Nile Virus (WNV), and Japanese Encephalitis Virus (JEV). The heat map shows the overlapping genes among the top 10 hits from 9 different CRISPR studies (C1 to C9) and their ranks based on the phenotypic significance (with 1 (Red) being the best to 10 (Yellow) being the last). Boxes colored in white denote that the gene is not present in the filtered dataset. The last column represents the number of studies in which the gene is present. Studies used in the analysis: C1 [53], C2 [54], C3 [55], C4 [56], C5 [57], C6 [58], C7 [59], C8 [60], C9 [61].

To probe for common genetic factors enriched among various CRISPR KO screens, we analyzed the overlapping genes from the top 10 hits from nine different screens (carried out for DENV, ZIKV, WNV, and JEV) and checked for intersections (Figure 2).

Several ER-associated proteins involved in membrane remodeling, protein stabilization, folding, and degradation emerged as common genetic factors for various viruses. Some of the important viral–host interactions uncovered by these studies are discussed in detail below:

### 3.1. Virus Receptors and Attachment Factors

Independent CRISPR Screens have revealed various cell surface molecules utilized by flaviviruses such as Zika, Dengue, and West Nile virus to enter the cell. These include heparan sulfate proteoglycans (HSPG) and TAM (e.g., Tyro3, Axl, and Mer) family receptor tyrosine kinases that interact with the envelope protein of the virus [58]. Genes associated with heparin sulfation (NDST1 and EXT1) have also been identified [58]. A recent CRISPR KO study performed in glioblastoma stem cells revealed integrin αvβ5 as an internalization factor for ZIKV. The authors further demonstrated that using an αvβ5 blocking antibody or two chemical inhibitors (SB273005 and cilengitide) brings down the ZIKV infection and alleviates ZIKV-induced pathology in human neural stem cells and mouse brains [67]. Another study by Marceau et al. in 2016 unraveled some receptors cardinal for Hepatitis C Virus (HCV) to enter hepatocytes. These include CD81, occludin (OCLN), and claudin 1 (CLDN1) [53]. Zhao et al. carried out a CRISPR KO screen on porcine kidney cells challenged with JEV and recognized many genes associated with heparan sulfate proteoglycans (HSPGs) and their metabolism. The study highlighted many potentially vulnerable targets for developing breeding technologies to combat and prevent JEV disease in pigs [61].

### 3.2. Viral Translation and Insertion into ER Membrane

**Figure 3:**
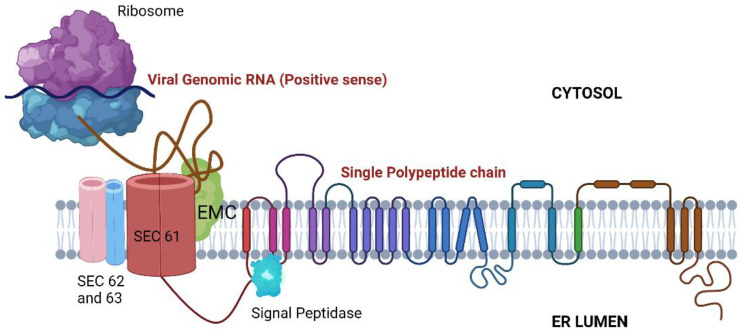
ER-associated proteins are involved in viral RNA translation, stabilization, and folding. SEC61: Translocon; EMC: Endoplasmic reticulum membrane protein complex.

Post uncoating, the viral RNA is translated by host ribosomes. Several proteins have been shown to interact with the nascent polypeptide for its proper folding, insertion into the ER membrane, and further processing (Figure 3):

- Signal Peptidase Complex (SPCS): After being translated, the flavivirus polyprotein is inserted into the ER membrane as a single multipass protein and cleaved by viral and host proteases, including the host signal peptidase complex (SPCS). Knocking out SPCS1, a significant component of the SPCS, ablated the replication of all flaviviruses but not that of the unrelated RNA viruses, suggesting that it is needed for flavivirus replication specifically. Mechanistic studies revealed that the SPCS1 is involved in the cleavage of the polyprotein’s structural proteins prM and E [60].
- Translocon-associated protein complex (TRAP): The SRP ribonucleotide complex recognizes and binds to a hydrophobic transmembrane region of the nascent polypeptide, arrests translation, and brings the ribosome to a translocon where translation continues. Since the translated polyprotein contains several transmembrane domains that need to be appropriately integrated into the ER membrane, the host SRP-translocon pathway proteins such as SEC61A1 and SEC63 also showed up in several CRISPR Screens [53,60,64,68] and in an RNAi Screen [66]. Additionally, several protein-protein interaction studies have revealed interactions between ZIKV/DENV NS4A and SEC62, SEC61γ, and SRPR; NS4A/2B and SEC61β; and NS4B with SEC61α [69,70]. Interestingly, pharmacological modulation of this complex has been shown to inhibit DENV and ZIKV replication [70,71].
- Endoplasmic-reticulum-associated protein degradation (ERAD) Pathway: ERAD is a protein quality control mechanism that recognizes incorrectly folded proteins in the ER lumen. These proteins are then retro-translocated through the ER membrane to the cytosol to be targeted for proteasomal degradation. Certain components of the classical ERAD machinery, especially the ones that form the retro-translocation complex, were shown to be essential for infectious virus particle formation and virus-induced cell death for DENV, ZIKV, JEV, and WNV. These include proteins such as SEL1L, derlin 2 (DERL2), and ubiquitin-conjugating enzyme E2 J1 (UBE2J1). Knocking out these genes conferred robust protection against WNV-induced cell death. Remarkably, WNV replication was unaffected. Thus, these factors have been speculated to be the chief drivers of WNV-induced cell death [59].
- The Endoplasmic reticulum membrane protein complex (EMC): EMC is an evolutionarily conserved complex responsible for stabilizing and helping in the insertion of multipass membrane proteins in the ER. Several genetic screens have independently shown the EMC proteins to be essential for correct viral protein insertion into the ER membrane [53,58,59,68]. A 2019 study suggested that biogenesis and co-translational stabilization of DENV and ZIKV multipass proteins NS4A and NS4B rely on the interaction with EMC components [72]. The authors used a dual-fluorescence reporter system to map the hydrophobic transmembrane regions of NS4B utilized for the interaction with the EMC complex. An independent study showed a very prominent loss of replication of DENV, ZIKV, and YFV upon knocking out protein complex EMC4. Interestingly, there was no effect on the replication of WNV. The authors speculated that this difference could be because Culex mosquitoes, rather than Aedes, primarily transmit WNV. To support this vector-specific hypothesis, they also interrogated the DENV titer in Aedes mosquito midguts, which was found to be depleted post-siRNA-mediated targeting of EMC2/3/4. All in all, the study suggested that the EMC is a critical host factor utilized by Aedes-transmitted flaviviruses [73].
- Additionally, two subunits of the endoplasmic reticulum (ER) resident dolichol-phosphate mannose synthase (DPMS) complex were identified as host dependency factors for DENV and ZIKV. The DPMS complex catalyzes the synthesis of dolichol-phosphate mannose (DPM), which serves as a mannosyl donor in pathways leading to N-glycosylation, glycosylphosphatidylinositol (GPI) anchor biosynthesis, and C- or O-mannosylation of proteins in the ER lumen. This DPMS complex was shown to be required for optimal viral RNA amplification and proper glycosylation and folding of viral structural proteins prM and E [55].

### 3.3. Formation of Replication Complexes (RCs) and Viral RNA Synthesis

**Figure 4:**
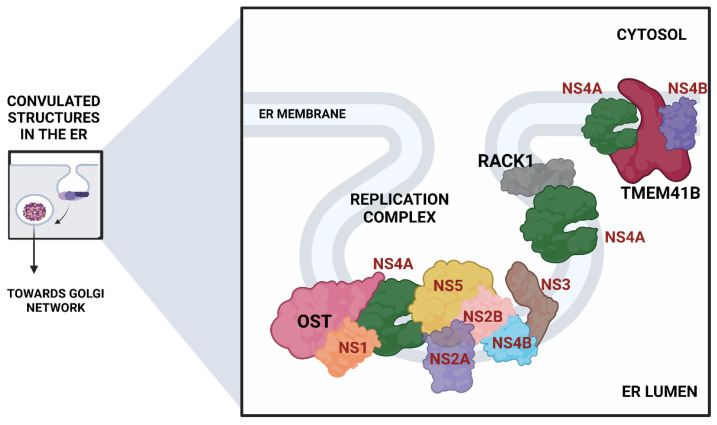
ER remodeling proteins associated with the formation of the viral replication complex. (Viral proteins are shown in red).

The translated viral proteins assemble in the form of a replication complex in close association with several ER-resident host protein complexes. Many CRISPR screens mapping host factors for DENV and ZIKV uncovered proteins involved in the formation of the oligosaccharyltransferase (OST) complex, the endoplasmic reticulum membrane protein complex (EMC), and components of the ER-associated protein degradation (ERAD) pathway (Figure 4).

- The Oligosaccharyltransferase (OST) complex: The OST complex is associated with N-linked glycosylation of host proteins in mammalian cells. Interestingly, different flaviviruses exhibit different dependencies on the two OST complex catalytic subunits: STT3A and STT3B. While the STT3A complex is needed for the co-translational N-linked glycosylation of the majority of the glycoproteins, the STT3B complex is essential for the co-translational or post-translational glycosylation of acceptor sites that have been skipped by the STT3A complex [74]. The OST complex was shown to be necessary for the viral RNA synthesis but not for the entry and translation. Both complexes were individually required for the replication of DENV. However, ZIKV replication was shown to be exclusively dependent on the STT3A complex, pointing out divergent virus-host interactions. Knocking out OST complex component STT3A abrogated the replication of YFV, WNV, and JEV as well. However, these replication defects were rescued by the expression of catalytically dead STT3A mutants, suggesting that the ability of OST complex to glycosylate proteins is not required for flavivirus replication. Additionally, physical interactions between flavivirus replication complex members NS1, NS2B, NS3, and NS4B and OST Complex in the ER suggest that the OST complex might act as a scaffold to orchestrate the assembly of the viral replication complex [53]. Lin et al. employed the same genome-wide CRISPR KO approach and extended this work to show that the oxidoreductase activity of the OST complex subunit MAGT1 was essential for DENV propagation. They further showed that the expression of MAGT1 depends on the presence of STT3B but not on its catalytic activity. MAGT1 was also associated with DENV NS1 and NS4B proteins during viral infection [54]. Collectively, these two studies suggested that the OST complex not only interacts physically with the replication complexes but is also engaged in post-translationally modifying and stabilizing the viral non-structural proteins associated with the complex.
- In another interesting genome-wide CRISPR KO study, Transmembrane Protein 41B (TMEM41B) was shown to be required for infection and replication of several mosquito-borne and tick-borne flaviviruses, making it a pan-flavivirus host factor. Based on mechanistic studies, the authors proposed a model whereupon flavivirus entry and subsequent translation of the viral polyprotein; this protein, TMEM41B, is recruited to the ER membrane together with viral proteins NS4A and NS4B, which are involved in inducing membrane curvature so that replication complexes (RCs) can form and make a protected environment for viral genome replication. The study also showed how the absence of TMEM41B leads to the formation of poor RCs, which ultimately causes the dsRNA replication intermediates to become exposed to innate immunity pattern recognition receptors (PRRs) in the host cell. This recognition and activation of innate immune responses lead to the abortion of the infectious replication cycle [68].
- Another significant flavivirus host factor is the Receptor for Activated C Kinase 1 (RACK1) protein. This protein has functions correlated with protein shuttling, anchoring, stabilization, and mediating specific cellular pathways through protein interactions. A recent CRISPR KO screen in Huh7 cells found that silencing of RACK1 affected the replication of several flaviviruses, including ZIKV, DENV, and WNV but not YFV. They utilized a Renilla luciferase DENV replicon to proclaim that RACK1 specifically played a role in viral genome replication rather than viral entry or translation. The authors used a replication-independent expression system to delineate the mechanism that induces the formation of RCs in the ER without virus infection. RACK1 silencing was shown to limit the organization of these structures in the ER membrane [56].
- Apart from these pathways and complexes, FAD biosynthesis, catalyzed by riboflavin (vitamin B2), kinase (RFK), and FAD synthase (FLAD1), was shown to be critical for the synthesis of HCV RNA. ELAVL1, an RNA-binding protein that binds to host mRNAs and increases their stability [75], was shown to attach to the 3′ UTR of HCV RNA to aid its replication via circularization [53]. Significantly, a protein called Cyclophilin A (CYPA) that has been shown previously to interact with HCV replication protein NS5A was also enriched (3). Some host cyclophilin inhibitors have shown promising effects in curing HCV infection in both in vitro and in vivo settings and have advanced to phase II/III clinical trials [76]. This study on cyclophilin inhibitors also highlights how targeting the host factors instead of viral factors is associated with the reduced emergence of resistance [76]. This is important because HCV exhibits a brisk mutation rate as an RNA virus, and a single mutation in the viral target can render the antiviral ineffective.

## 4. CRISPR Screens to Identify Anti-Flavivirus Host Factors

In addition to CRISPR KO screens, some studies have tried decoding antiviral genes using genome-wide CRISPR activation screens. Most of the high-ranking hits from one such study included interferon-stimulated genes (ISGs) such as interferon lambda 2 (IFN-λ2) and interferon alpha-inducible protein 6 (IFI6). They were shown to provide high levels of protection from the early stages of ZIKV infection. Furthermore, the identified hits were very significantly induced in ZIKV-infected placenta explants. This study is an exciting example substantiating CRISPR activation screens as a tool to decode antiviral host factors [62]. Richardson et al. used CRISPR KO screening to identify genes that regulate interferon (IFN) response to flavivirus infection [77]. In this study, the cells were treated with a high dose of IFN-α before performing a CRISPR screen to decode host antiviral factors that make cells susceptible to infection when knocked out. Several members of the IFN-α signaling pathway, namely, IFNAR1, IFNAR2, IRF9, and ISG effector gene IFN-α-inducible protein 6 (IFI6), were identified as factors with the highest antiviral activity. Further experiments showed that IFI6 prophylactically protects the uninfected cells and prevents the formation of virus-induced invaginations in the ER membrane, and impairs viral replication. Interestingly, this protein IFI6 had a faint effect on other mammalian RNA viruses, including HCV, which replicate in double membrane RCs that protrude outwards instead of inwards from the ER membrane [77].

## 5. Conclusions and Prospective

This review describes the main types, principles, and steps of pooled CRISPR KO screens. Since their application at the genome-scale, CRISPR tools have contributed tremendously to fundamental and translational studies. Various Cas proteins and sgRNAs have been used to develop gain-of-function CRISPRa and loss-of-function CRISPRi tools. Studying emerging, re-emerging, and persistent viruses such as those belonging to the *Flaviviridae* family and their crosstalk with host cells is crucial for discerning their biology and host response. In addition, this information can be exploited to formulate therapeutic molecules or improve existing strategies against flavivirus disease and transmission. It is noteworthy that there are subtle differences in data sets from independent CRISPR screens for the same viral infections. These can arise due to differences in experimental setups, virus strains, the target cell line used, or types of CRISPR screening. However, there are studies with typically overlapping gene hits for the same virus challenge. This reproducibility is remarkable and is the principal advantage of CRISPR-Cas9 technology. Moreover, with the advent of combinatorial CRISPR screens, one can easily define the most effective combinations of host factors or cellular signaling pathways that can be targeted to impede viral infections [78]. Although not discussed here, it is worth noting that additional virus-host interactions, such as RNA–RNA, protein-protein, and RNA–protein interactions, also contribute remarkably to our conception of flavivirus replication and pathogenesis [70,79,80]. In the recent past, there has been significant development in the CRISPR field, with the development of more precise Cas enzymes with single base editing abilities [81] or others with RNA targeting activities [82]. These tools are yet to be used extensively to examine flavivirus–host interactions.

Furthermore, since flaviviruses persist in arthropod vectors, it becomes vital to comprehend how host factor interactions overlap or differ between the insect vector and human hosts. This information could uncover fundamental interactions shared across diverse hosts and certain cellular factors that can be used as targets of drug/chemical or genetic manipulation in vector species to reduce virus dissemination. To date, there has been no genome-wide CRISPR screen to decode the mosquito-flavivirus–host factors. Some studies, however, have tried to systematically uncover insect host factors required for DENV-2 propagation by employing a genome-wide RNA interference screen in Drosophila melanogaster cells [64]. Such studies highlight the conservation of host dependency factors between dipteran and human hosts and reveal genetic factors that can be targeted to control infection in both the insect vector and the mammalian host [64]. As sequencing and gene annotations of *Aedes* mosquito and tick species improve, carrying out genome-wide screens to uncover flavivirus–host factors for these species becomes more feasible.

In conclusion, genome-wide CRISPR screens provide a powerful tool to decode the flavivirus–host interactions. We believe that future advances in applying this technology to other hosts and cell types will shed light on how flaviviruses evolve to exploit and subvert host functions. Such understanding will go a long way in uncovering the novel aspects of flavivirus biology and help develop better therapeutics and strategies for medical interventions and vector control.

## Figures and Tables

**Table 1:** Summary of Genome-Wide Genetic Screens to uncover host dependency and restriction factors for flaviviruses.

Type of Genetic Screen	Authors and References	Cell Line	Virus Used for Challenge
**CRISPR** **KO** **Screens**	**Caleb D. Marceau et al. (2016)** [53]	HuH 7.5.1	DENV
	**David L Lin et al. (2017)** [54]	Huh 7.5.1	
	**Athena Labeau et al. (2020)** [55]	Haploid HAP1	
	**Byron Shue et al. (2021)** [56]	Huh 7.5	ZIKV
	**Yun Li et al. (2019)** [57]	Human pluripotent stem cell (hPSC)-derived neural progenitors (NPs)	
	**George Savidis et al. (2016)** [58]	Huh 7.5	
	**H. Ma et al. (2015)** [59]	293FT cells	WNV
	**Rong Zhang et al. (2016)** [60]	293T-Cas9 cells	
	**Changzhi Zhao et al. (2020)** [61]	Porcine kidney-15 (PK-15) cells	JEV
	**H.-Heinrich Hoffman et al. (2020)**	B3GALT6-deficient human haploid (HAP1) cells	YFV and ZIKV
**CRISPRa** **Screen**	**Anna Dukhovny et al. (2019)** [62]	Huh-7	ZIKV
	**Anh Phuong Luu (2021)** [63]	Human STAT1−/− fibroblasts	
**Haploid Genetic Screens**	**Caleb D. Marceau et al. (2016)** [53]	Haploid HAP1	DENV
**siRNA**	**George Savidis et al. (2016)** [58]	HeLa	DENV
	**October M. Sessions et al. (2009)** [64]	Huh-7	
	**Caroline Le Sommer et al. (2012)** [65]	Huh-7	YFV
	**Manoj N Krishnan et al. (2008)** [66]	HeLa	WNV

## Data Availability

Not applicable.
